# Spontaneous Rupture of Uterine Artery in a 14-Week Pregnant Woman

**DOI:** 10.1155/2016/9524250

**Published:** 2016-11-06

**Authors:** João Paulo Mancusi de Carvalho, Luciano Augusto de Carvalho Severo, Maria Helena Mancusi de Carvalho, Marina de Paula Andres, Mariano Tamura Vieira Gomes, Sergio Podgaec

**Affiliations:** Department of Obstetrics and Gynecology, Hospital Israelita Albert Einstein, São Paulo, SP, Brazil

## Abstract

We report a case of uterine artery rupture in a woman at 14 weeks' gestation who presented with abdominal pain, tachycardia, and hypotension and underwent a diagnostic laparoscopy. During this procedure, a spontaneous rupture of the left uterine artery was diagnosed and the surgery was converted into a laparotomy. The artery was bound to its origin and to its distal uterine portion. The patient exhibited excellent postoperative recovery and was discharged two days after the surgery. The pregnancy continued without other maternal or fetal complications, and the patient delivered a healthy newborn via cesarean section at 39 weeks of gestation.

## 1. Introduction

During pregnancy there are specific physiological changes that can leave pregnant women more vulnerable to vascular changes due to hormonal alterations. Spontaneous rupture of the uterine artery is an extremely rare phenomenon with high morbidity and mortality [1]. There are few reports of this situation in the medical literature mainly in the first trimester of pregnancy [1, 2].

## 2. Case Report

A 30-year-old pregnant patient at 14 weeks' gestation, who was in her second pregnancy with a previous cesarean delivery, went to her primary care physician with mild abdominal pain and nausea. After receiving fluids and endovenous analgesics, resulting in an improvement of symptoms, she was discharged. Twelve hours after the onset of pain, she came to the first-aid post with diffuse abdominal pain, malaise, and reports of syncope. On admission, she exhibited mucocutaneous pallor, tachycardia (108 bpm), mild hypotension (BP 90/60 mmHg), moderate hypogastric pain, decreased bowel sounds, and a positive rapid decompression test. The uterus was palpable 2 cm above the pubic symphysis and the fetal heartbeat was 140 bpm. Secondary exams showed hemoglobin at 5.3 g/dL and hematocrit level of 16.7%. An ultrasound revealed a large amount of free fluid in the abdomen, suggesting the presence of hemoperitoneum.

**Figure 1:**
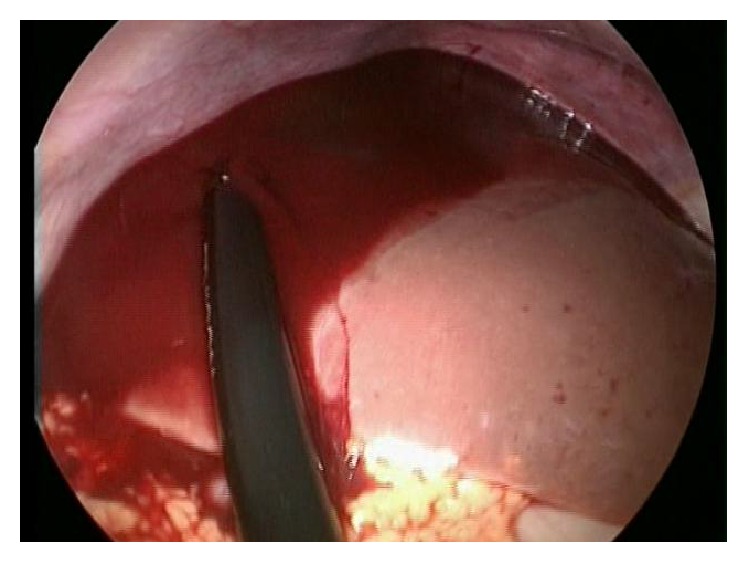
Diagnostic laparoscopy: blood identification in the right hypochondrium (liver).

**Figure 2:**
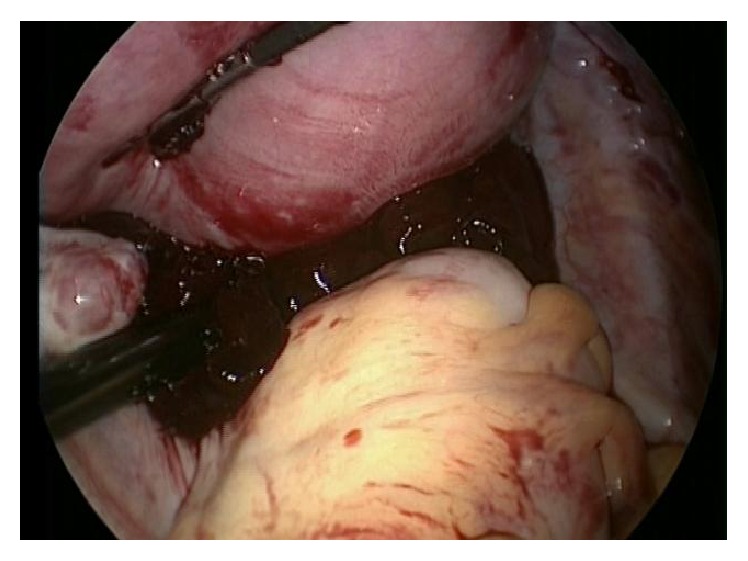
Diagnostic laparoscopy: clot identification in the Douglas pouch.

**Figure 3:**
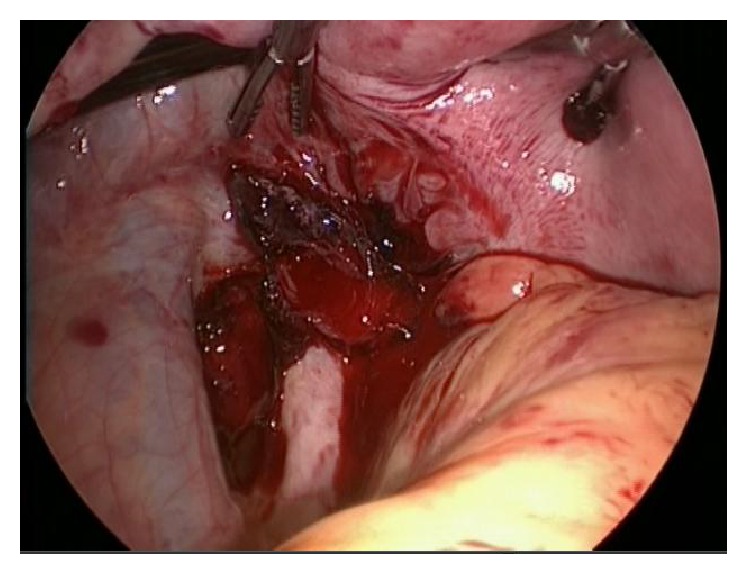
Diagnostic laparoscopy: identification of active arterial bleeding by topography of the left uterine vessels.

The patient was immediately taken to the operating room for a diagnostic laparoscopy. During the inventory of the abdominal cavity, about 2.5 liters of blood was aspirated (Figures 1 and 2) and after careful inspection, active arterial bleeding in the left uterine vessel topography was detected (Figure 3). Due to the uterine size and technical difficulty, the most feasible surgical option was conversion to Pfannenstiel laparotomy, followed by dissection of the left retroperitoneal space with identification and isolation of the left iliac vessels and ureter. Rupture of the left uterine artery was observed, after which it was connected to its origin and to its distal portion (close to the uterus) obtaining full bleeding control.

Fetal viability was evaluated by obstetric ultrasound and remained preserved. After the transfusion of three units of packed red blood cells, the patient demonstrated an excellent recovery and was discharged two days after surgery.

The pregnancy continued without complications and childbirth occurred electively at 39 weeks of pregnancy via cesarean section. The newborn weighed 2900 g, with an Apgar score of 10 at the 5th minute, and was discharged with her mother on the third postpartum day.

## 3. Discussion

Rupture of pelvic vessels during pregnancy is a rare and potentially fatal event, resulting in maternal mortality rates of up to 3.6% [3]. By 2006, only 42 cases were reported and occurred mainly, in the second and third trimester of pregnancy, although rupture may also occur up to three weeks after delivery [4–6]. This report shows a much earlier presentation compared to other cases described in the literature, since the patient was a pregnant woman at 14 weeks' gestation.

Blood loss caused by vascular abdominal lesions during pregnancy can occur by the rupture of different vessels, such as the hepatic artery (secondary to HELLP syndrome), or the renal, splenic, uterine, and ovarian vessels [3]. This vascular weakness can occur because the initial maintenance of pregnancy depends on progesterone action, which provides a decrease in peripheral vascular resistance and in blood pressure, resulting in an increase of the number and diameter of the uterine vessels [7]. Moreover, the growing uterus compresses pelvic vessels and the inferior vena cava, disturbing venous return and causing a threefold increase in the venous pressure in pelvic vessels, which may predispose their rupture [2].

Other conditions that can contribute to arterial rupture are pelvic endometriosis (due to local inflammation), increased intra-abdominal pressure secondary to stress when coughing, sexual intercourse, and expulsion of the fetus [1, 5, 8, 9]. In women with congenital malformations and vascular degenerative processes, the formation of aneurysms can also be a cause of rupture of the uterine and pelvic arteries [10].

This clinical condition is nonspecific and presents as abdominal pain of sudden onset with a gradual increase in pain as blood loss occurs, followed by mucocutaneous paleness, tachycardia and arterial hypotension. The interval between the rupture of the uterine vessels and the occurrence of hemodynamic disturbances can be up to 72 hours [11]. Laboratory findings include a gradual reduction of hematological parameters (hemoglobin, hematocrit, and number of red blood cells) and the presence of free fluid in the abdominal cavity observed via ultrasound [7]. Magnetic resonance angiography and arteriography can be used in selected cases for diagnosis and treatment, with selective embolization of the injured artery [12]. The evaluation of fetal viability is essential and helps to exclude obstetric causes of bleeding, such as placenta previa, and abdominal pain due to active labor.

The best approach in patients with suspected hemoperitoneum during pregnancy is to perform a laparoscopy. Differential diagnoses include ovarian cyst rupture, ectopic pregnancy, splenic rupture, and rupture of a splenic artery aneurysm [3, 13].

Laparoscopy allows for a proper assessment of the whole abdomen, which would otherwise only be possible with a midline laparotomy using a single incision extending from the xiphoid process to the pubic symphysis. Depending on the surgeon's experience and the clinical condition of the patient, many of the causes of bleeding can be repaired using laparoscopy, leading to less surgical trauma and better recovery of pregnant women [13]. In the case presented herein, it was not possible to repair the injury by laparoscopy, mainly due to the enlarged, gravid uterus. However, starting with a laparoscopic procedure was essential for greater diagnostic accuracy and allowed for the best possible incision with a favorable aesthetic result and decreased morbidity in relation to the incision.

Roger et al. [14] and Vellekoop et al. [15] reported cases for two patients at 25 and 27 weeks' gestation that demonstrated a spontaneous rupture of uteroovarian vessels, treated with vascular suture via laparotomy. The pregnancies reached the 38-week point and resulted in vaginal births with healthy newborns.

However, according to reports described by Dubuisson et al. [4], there were three cases in which similar patients were in gestational periods between 30 and 32 weeks, and the fetuses did not survive, possibly due to late diagnosis of the uterine vessel rupture, prematurity, or high maternal blood loss. Early diagnosis of patients with rupture of uterine artery is critical, since the pressure of the gravid uterus on the uterine vessels, the increase of maternal blood volume during pregnancy, and other compensatory mechanisms delay the onset of hypovolemia symptoms, potentially causing high maternal and fetal morbidity [16].

In conclusion, the rupture of uterine vessels during pregnancy is a rare but dramatic event. Pregnant women who present with acute abdominal pain should undergo laboratory tests followed by ultrasound and in cases of suspected hemoperitoneum, a laparoscopy should be promptly carried out as a first line intervention.
